# Comparison of therapeutic effects of different acupuncture and moxibustion therapies in the treatment of peripheral facial nerve paralysis: A protocol for systematic review and network meta-analysis

**DOI:** 10.1097/MD.0000000000028926

**Published:** 2022-04-01

**Authors:** Ming Li, JianGuo Ruan, HuaJun Zhang, JiuLong Wu, YuJuan Wang, Shanshan Zhu

**Affiliations:** a Nanjing Drum Tower Hospital, The Affiliated Hospital of Nanjing University Medical School, Nanjing, China; b Nanjing Xuanwu Hospital, Nanjing, China.

**Keywords:** acupuncture and moxibustion, network meta-analysis, peripheral facial nerve paralysis, protocol

## Abstract

**Background:**

Previous evidences show that acupuncture and moxibustion therapy has positive effects on peripheral facial nerve paralysis (PFP), but there are many acupuncture treatments based on meridian theory, and there are differences in the efficacy of each program. This study will compare the clinical efficacy of different acupuncture treatments for PFP through mesh meta-analysis.

**Methods:**

Randomized controlled trials of acupuncture therapy in the treatment of PFP are going to be retrieved from 8 Science databases including CNKI, Wanfang, VIP and Chinese Biomedical Science, PubMed, Embase, Web of Science and the Cochrane Library from establishment to January 2022. We will use the Cochrane Risk Bias Assessment Tool to assess the quality of the studies and the grading of recommendation assessment, development and evaluation method to assess the strength of the evidence. All data analyses will be performed by Revman5.3, Gemtc 0.14.3, and Stata 14.0.

**Results:**

This study will evaluate the efficacy of different acupuncture treatments for PFP by evaluating clinical efficacy rate, facial nerve function score, facial disability score scale, facial electromyography, adverse reactions, etc, and further explore the mechanism of action of each therapy.

**Conclusion:**

This study will provide a reliable evidence-based basis for selecting the best acupuncture treatment for PFP.

**Trial registration:**

Open science framework registration number: DOI 10.17605/OSF.IO/XQRK9

## 1 Introduction

Peripheral facial nerve paralysis (PFP), also known as facial neuritis, is facial muscle paralysis caused by nonspecific inflammation of the facial nerve in the facial nerve tube, resulting in facial expression muscle dysfunction.^[1]^ It is estimated that the incidence of facial nerve palsy in the world is 20∼25 cases per 100,000 people per year,^[2]^ whose onset can occur at any age, with no seasonal differences.^[3]^ PFP can cause drooping eyebrows, incomplete eyelid closure, dry eyes, hyper-hearing, impaired taste, and mouth closure problems that can seriously affect facial function and appearance.^[4]^ After systematic treatment, most patients recover completely, but about 15%∼30% are reported to be left with sequelae of varying degrees.^[5]^ Failure to recover or incomplete recovery of facial nerve function will bring enormous social and psychological pressure to patients, seriously affect social activities and quality of life, and the occurrence of facial paralysis sequelae will cause long-term impact.^[3]^ Therefore, how to further improve the effective cure rate of PFP is of great significance.

At present, the treatments in western medicine mainly include corticosteroids, antivirus, nerve nutrition and vitamin B drugs.^[6,7]^ However, these drugs all have different degrees of side effects, such as long-term use of hormone drugs can inhibit the immune function of the body, cause gastrointestinal irritation and gastrointestinal bleeding, glaucoma, and femoral head ischemic necrosis, etc.^[3]^ Antiviral drugs may cause nausea, vomiting and other digestive symptoms.^[8]^ Facial nerve decompression can also be performed in patients with insignificant improvement in facial nerve function, but the therapy is still controversial and not easily recognized by patients.^[9]^

Acupuncture and moxibustion based on meridian theory is widely used in PFP as a complementary and alternative therapy, which has the characteristics of safety, effectiveness and low cost.^[10–12]^ However, there are many forms of acupuncture, such as needle acupuncture, electroacupuncture, and moxibustion. Although they are all based on the meridian theory of traditional Chinese medicine, the methods of use are different. For example, compared with steroids, warm acupuncture can improve the therapeutic effect of PFP and improve the facial nerve function.^[13]^ Needle acupuncture can improve PFP better than steroid, but the recovery speed is the same.^[14]^ In addition, the combined use of different acupuncture forms has also appeared in clinical practice, such as needle acupuncture combined with acupuncture, electroacupuncture combined with moxibustion and so on.^[15,16]^ Existing evidence shows that various forms of acupuncture and moxibustion have advantages over western drugs in the treatment of PFP, but the lack of direct comparison between different forms of acupuncture and moxibustion makes it impossible to judge which form of acupuncture and moxibustion is better in the treatment of PFP. Network meta-analysis is a method developed from traditional meta-analysis, which can compare the differences between 2 therapeutic measures through a common control when there is no direct comparison, so as to compare and rank the advantages and disadvantages of multiple clinical interventions.^[17]^ Therefore, network meta-analysis is used in our study to compare the efficacy of different acupuncture and moxibustion therapies on PFP, in order to provide evidence-based evidence for the selection of the optimal acupuncture treatment in clinical treatment of PFP.

## 2 Methods

### 2.1 Protocol register

This study was conducted according to the preferred reporting items for systematic review and meta-analysis protocols for network meta-analysis guidelines.^[18]^ Moreover, it has been registered on open science framework on January 13, 2022 (Registration number: DOI 10.17605/OSF.IO/XQRK9).

### 2.2 Ethics

Since the program does not require patient recruitment and collection of personal information, it requires no approval of an ethics committee.

### 2.3 Eligibility criteria

(1) Study subjects: the patients were diagnosed with PFP, and the original study had clear and authoritative diagnostic criteria of traditional Chinese medicine or Western medicine for PFP. The gender, age and race of the patients were not limited.
(2) Study type: randomized controlled trial, with or without blindness, in Chinese and English.
(3) Intervention measures: the treatment group was treated with different forms of acupuncture based on meridian theory, including conventional acupuncture, warm acupuncture, electroacupuncture, fire acupuncture, moxibustion, auricular acupoint pressing, acupoint embedding and acupoint injection, which could be used alone or in combination; the control group was given western medicine or placebo.
(4) Exclusion criteria:
  i. Duplicate published studies;
  ii. The treatment group included traditional Chinese medicine therapy, such as Chinese medicine, massage, etc;
  iii. Reviews, experience presentations, conference articles, reviews or case reports;
  iv. Studies in which data were incorrect or incomplete and complete data could not be obtained by contacting the authors.

### 2.4 Outcome indicators

(1) Main outcome indicators: clinical efficacy rate; facial nerve function score (eg, House–Brackmann Grading Scale, Facial Nerve Grading System 2.0).^[19]^
(2) Secondary outcome indicators: other facial grading scales (eg, Sunnybrook Facial Grading System,^[20]^ Portmann Score, and facial Disability Index Score); facial electromyography;^[21]^ adverse reactions.

### 2.5 Search strategy

**Figure 1:**
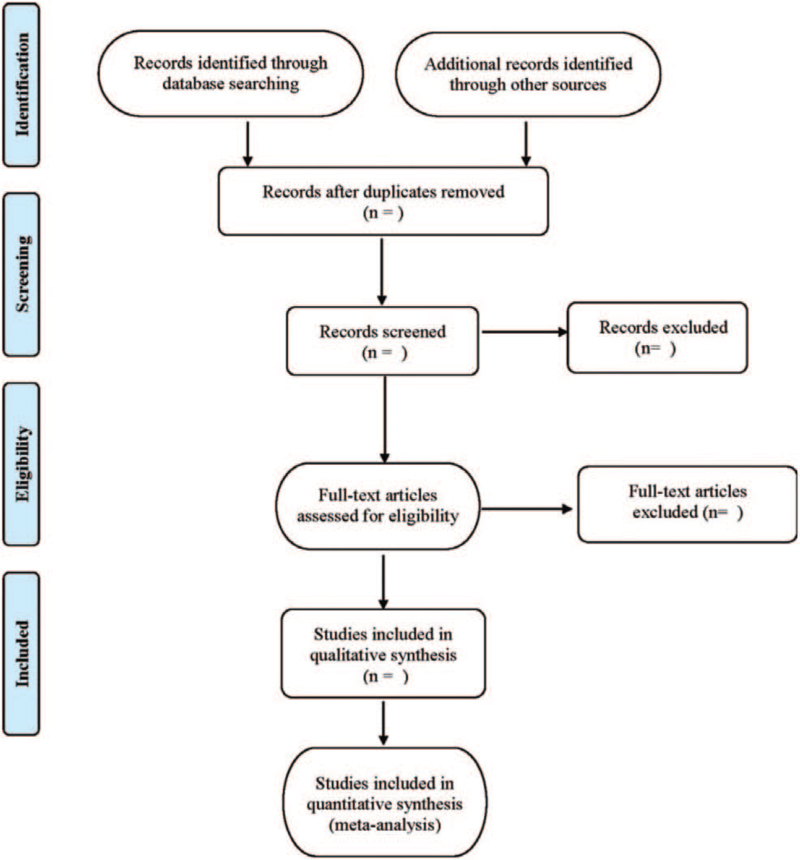
Flow diagram.

Randomized controlled trials on acupuncture treatment of PFP will be independently searched by 2 researchers, and the search time is set to January 2022. The search database includes: PubMed, EMBASE, Web of Science, Cochrane Library, CNKI, WanFang Data, VIP, China Biomedical Literature Database. Chinese search terms are “zhen ci” (acupuncture), “dian zhen” (electroacupuncture), “wen zhen jiu” (warm acupuncture), “huo zhen” (fire acupuncture), “ai jiu” (moxibustion), “xue wei mai xian” (acupoint embedding), “zhou wei xing mian tan” (peripheral facial nerve paralysis), “mian shen jing yan” (facial neuritis), etc. English search terms are “acupuncture”, “eletroacupuncture”, “warm needle”, “fire needle”, “moxibustion”, “acupoint catgut embedding”, “acupoint injection”, “Peripheral facial nerve paralysis”, “PFP”. The included literature will be independently screened by 2 researchers according to the inclusion and exclusion criteria. If there is any difference of opinion, it will be decided after consultation with the third researcher. PubMed search strategy is shown in Figure 1.

### 2.6 Data screening and extraction

Literature screening and data extraction will be carried out independently by 2 researchers and cross-checked. Disagreements will be discussed and decided with a third researcher. The extraction content includes the basic information of the included literature (first author, publication date, research title); relevant information of the treatment group and the control group in the literature (including the number of cases, total cases, gender, age, intervention measures, course of treatment, and outcome indicators); design types and quality evaluation information of the included literature. The literature screening process is shown in Table 1.

**Table 1:** Search strategy in PubMed database.

Number	Search terms
#1	Acupuncture [MeSH]
#2	Acupuncture [Title/Abstract]
#3	Pharmacopuncture [Title/Abstract]
#4	Electro-acupuncture [Title/Abstract]
#5	Warm needle [Title/Abstract]
#6	Fire needle [Title/Abstract]
#7	Blood-letting puncture [Title/Abstract]
#8	#1 OR #2 OR #3 OR #4 OR #5 OR #6 OR #7
#9	Facial paralysis [MeSH]
#10	Facial palsy [Title/Abstract]
#11	Hemifacial paralysis [Title/Abstract]
#12	Lower motor neuron facial palsy [Title/Abstract]
#13	Peripheral facial paralysis [Title/Abstract]
#14	Peripheral facial nerve paralysis [Title/Abstract]
#15	Facial paresis [Title/Abstract]
#16	Bell palsy [Title/Abstract]
#17	#9 OR #10 OR #11 OR #12 OR #13 OR #14 OR #15 OR #16
#18	#8 AND #17

### 2.7 Literature quality assessment

The included studies will be evaluated for risk bias according to the quality evaluation tools recommended by Cochrane 5.1 Systematic Evaluation Manual,^[22]^ including: random sequence generation; allocation concealment; participant and personnel blinding; outcome assessment blinding; incomplete outcome data; selective reporting; and other bias. Two researchers will evaluate the above contents as “low risk”, “high risk”, and “unclear”, and cross-check the evaluation results. If there are differences, discuss them, and if no agreement can be reached, negotiate with the third researcher. Finally, RevMan5.3 will be used to draw a risk map of bias.

### 2.8 Statistical analysis

We will use Stata14.0 software to draw evidence network diagram to present the comparison between treatment programs. Mesh meta-analysis will be conducted using GeMTC14.3 based on Bayesian evidence framework. The effect value of dichotomous variables is represented by odd ratio, and the effect value of continuous variables is represented by mean different. The 95% confidence interval is used to represent the statistical analysis results. The Markov Chain Monte Carlo fitting consistency model will be used for Bayesian inference. Four chains are going to be used for simulation. The number of iterations is set as 50,000 (the first 20,000 for annealing and the last 30,000 for sampling). The potential Scale reduction factor will be used to reflect the convergence degree of the model. When potential Scale reduction factor is close to or equal to 1, it indicates that the data convergence is good and the obtained results are highly reliable.

### 2.9 Assessment of inconsistency

Node splitting analysis will be used to decompose mixed evidence into direct evidence and indirect evidence to evaluate model inconsistencies. If *P* > .05, there is no statistical significance, and consistency model is used for analysis; otherwise, inconsistency model is used for analysis. Stata14.0 will be used to calculate the surface under the cumulative ranking curve of different interventions, and the larger the surface under the cumulative ranking curve value, the better the therapeutic effect of the intervention. Finally, a comparative-correction map will be drawn to assess whether the small sample effect exists.

### 2.10 Assessment of publication bias

The comparison- adjusted funnel plots will be obtained with the specific ranking order to detect small sample size study effects and publication bias. All analyses will be conducted using R V.3.6.1 with the GeMTC package.

### 2.11 Evidence quality evaluation

Two researchers will use grading of recommendation assessment, development and evaluation to evaluate the quality of all comparative direct, indirect and mixed evidence.^[23]^ The evaluation includes risk of bias, indirectness, inconsistency, inaccuracy and publication bias. There are 4 levels of evidence quality: high, medium, low or very low. Two researchers will cross-check the results of the assessment, discuss any differences, and reach an agreement with a third researcher.

## 3 Discussion

So far, the etiology of PFP is still unclear, and the possible causes include cold stimulation/neurovascular ischemia, viral infection/reactivation, abnormal facial nerve anatomical structure, etc.^[24]^ Although PFP is not life-threatening, it brings heavy psychological burden to patients and seriously affects their quality of life. Therefore, effective treatment of PFP has always been a hot spot of medical attention.

In China, acupuncture has significant efficacy in the treatment of PFP and is widely used.^[11,25]^ A Cochrane Review on acupuncture treatment of PFP has concluded that acupuncture is beneficial to PFP without harmful side effects.^[26]^ Acupuncture can regulate sympathetic and parasympathetic nerves by stimulating afferent nerve fibers and their receptors, improve local blood circulation and nerve edema, and at the same time improve the excitability of neurons and promote the repair of injured neurons.^[27]^ However, there are also differences in the mechanisms of action between different acupuncture methods, which also lead to differences in efficacy. This study aims to directly or indirectly compare various acupuncture and moxibustion therapies through mesh meta-analysis, and sort them according to efficacy indicators, so as to screen out the best clinical treatment measures, in order to provide reliable evidence-based medical evidence for clinical practice.

However, there are some limitations in our study: due to the limitation of language retrieval, we only include Chinese and English literatures, which may cause selection bias; differences in disease type (course of disease, age, type, etc) and interventions (frequency, acupoint, course of treatment, etc) will increase the likelihood of heterogeneity. Nevertheless, we believe that the results of this study will help to find the best acupuncture and moxibustion treatment for PFP.

## Author contributions

**Data curation:** Ming Li, HuaJun Zhang.

**Funding acquisition:** Shanshan Zhu.

**Methodology:** Shanshan Zhu, JiuLong Wu.

**Software:** JianGuo Ruan, JiuLong Wu.

**Supervision:** HuaJun Zhang, JiuLong Wu.

**Writing – original draft:** JianGuo Ruan, YuJuan Wang.

**Writing – review & editing:** YuJuan Wang, Ming Li.

## References

[1] Peitersen E. Bell's palsy: the spontaneous course of 2,500 peripheral facial nerve palsies of different etiologies. Acta Otolaryngol Suppl 2002;4–30. 12482166

[2] Hong J Wu G Zou Y. Electroacupuncture promotes neurological functional recovery via the retinoic acid signaling pathway in rats following cerebral ischemia-reperfusion injury. Int J Mol Med 2013;31:225–31. 23129018 10.3892/ijmm.2012.1166

[3] Zhu YH Zheng XL Saina. Research progress and diagnosis status of Bell facial paralysis. Chin J Otol 2020;18:768–73.

[4] Fei J Gao L Li HH. Electroacupuncture promotes peripheral nerve regeneration after facial nerve crush injury and upregulates the expression of glial cell-derived neurotrophic factor. Neural Regen Res 2019;14:673–82. 30632508 10.4103/1673-5374.247471 PMC6352598

[5] Lj⊘stad U Økstad S Topstad T Mygland A Monstad P. Acute peripheral facial palsy in adults. J Neurol 2005;252:672–6. 15778908 10.1007/s00415-005-0715-1

[6] Baugh RF Basura GJ Ishii LE. Clinical practice guideline: Bell's palsy executive summary. Otolaryngol Head Neck Surg 2013;149:656–63. 24190889 10.1177/0194599813506835

[7] de Almeida JR Guyatt GH Sud S. Management of Bell palsy: clinical practice guideline. CMAJ 2014;186:917–22. 24934895 10.1503/cmaj.131801 PMC4150706

[8] Somasundara D Sullivan F. Management of Bell's palsy. Aust Prescr 2017;40:94–7. 28798513 10.18773/austprescr.2017.030 PMC5478391

[9] Kim J. Facial nerve decompression for Bell's palsy: an endless debate. Clin Exp Otorhinolaryngol 2019;12:331–2. 31575104 10.21053/ceo.2019.01515 PMC6787471

[10] Yu Z Shen M Shang W. Timing of acupuncture treatment in peripheral facial paralysis: a systematic review and meta-analysis. Comput Math Methods Med 2021;2021:4221955. 34956397 10.1155/2021/4221955 PMC8694981

[11] Zheng H Li Y Chen M. Evidence based acupuncture practice recommendations for peripheral facial paralysis. Am J Chin Med 2009;37:35–43. 19222110 10.1142/S0192415X09006631

[12] Wang WH Jiang RW Liu NC. Electroacupuncture is effective for peripheral facial paralysis: a meta-analysis. Evid Based Complement Alternat Med 2020;2020:5419407. 32328134 10.1155/2020/5419407 PMC7150689

[13] Zhang QQ Wang Y. Effect of warm acupuncture on peripheral facial paralysis. Clin J Tradit Chin Med 2017;29:222–4.

[14] Tong FM Chow SK Chan PY. A prospective randomised controlled study on efficacies of acupuncture and steroid in treatment of idiopathic peripheral facial paralysis. Acupunct Med 2009;27:169–73. 19942723 10.1136/aim.2009.000638

[15] Li HT Liu JH. Clinical observation on treatment of peripheral facial paralysis with acupuncture and pricking-cupping therapy. J Chin Integr Med 2005;3:18 69. 10.3736/jcim20050106 15644153

[16] Ying L Yan L Li-an L. Acupuncture and moxibustion for peripheral facial palsy at different stages: multi-central large-sample randomized controlled trial. Chin Acupunct Moxib 2011;31:289–93. 21528591

[17] Li L Tian JH Yao L. Statistical basis, hypothesis and quality evaluation of evidence in mesh meta-analysis. J Evid Based Med 2015;15:180–3.

[18] Hutton B Salanti G Caldwell DM. The PRISMA extension statement for reporting of systematic reviews incorporating network meta-analyses of health care interventions: checklist and explanations. Ann Intern Med 2015;162:777–84. 26030634 10.7326/M14-2385

[19] Lee HY Park MS Byun JY. Agreement between the facial nerve grading system 2.0 and the house-Brackmann grading system in patients with Bell palsy. Clin Exp Otorhinolaryngol 2013;6:135–9. 24069515 10.3342/ceo.2013.6.3.135 PMC3781225

[20] Neely JG Cherian NG Dickerson CB. Sunnybrook facial grading system: reliability and criteria for grading. Laryngoscope 2010;120:1038–45. 20422701 10.1002/lary.20868

[21] Bertozzi N Bianchi B Salvagni L. Activity evaluation of facial muscles by surface electromyography. Plast Reconstr Surg Glob Open 2020;8:e3081. 33173663 10.1097/GOX.0000000000003081 PMC7647650

[22] Cumpston M Li T Page MJ. Updated guidance for trusted systematic reviews: a new edition of the Cochrane Handbook for Systematic Reviews of Interventions. Cochrane Database Syst Rev 2019;10:Ed000142. 31643080 10.1002/14651858.ED000142 PMC10284251

[23] Puhan MA Schünemann HJ Murad MH. A GRADE working group approach for rating the quality of treatment effect estimates from network meta-analysis. BMJ (Clin Res ed) 2014;349:g5630. 10.1136/bmj.g5630 25252733

[24] Buizert A Verboon JC Wirtz PW. Bell's palsy: different doctor, different care? Nederlands tijdschrift voor geneeskunde 2018;162. 30211994

[25] Zhao YQ HL. Bibliometric analysis of acupuncture research through the Web of Science database from 1990 to 2019. Tradit Med Res 2021;6:9.

[26] Chen N Zhou M He L. Acupuncture for Bell's palsy. Cochrane Database Syst Rev 2010;2010:Cd002914. 10.1002/14651858.CD002914.pub5 PMC7133542 20687071

[27] Hontanilla B Marre D Cabello A. Masseteric nerve for reanimation of the smile in short-term facial paralysis. Br J Oral Maxillofac Surg 2014;52:118–23. 24148699 10.1016/j.bjoms.2013.09.017

